# Effects of BPD tendencies and subjective well-being on NSSI in adolescents with PTSD

**DOI:** 10.3389/fpsyt.2023.1152352

**Published:** 2023-06-15

**Authors:** Weixi Deng, Shu Yan, Yongjun Xu, Zhaoyuan Lu, Lianzhong Liu, Yang Zhou, Mo Chen

**Affiliations:** ^1^Affiliated Wuhan Mental Health Center, Tongji Medical College of Huazhong, University of Science and Technology, Wuhan, China; ^2^Department of Psychiatry, Wuhan Mental Health Center, Wuhan, China; ^3^Department of Psychiatry, Wuhan Hospital for Psychotherapy, Wuhan, China; ^4^Wuhan Dongfang Bode Psychiatric Hospital, Wuhan, China; ^5^School of Medicine, Jianghan University, Wuhan, China

**Keywords:** posttraumatic stress disorder, non-suicidal self-injury, personality characteristics, subjective happiness, adolescent, family function

## Abstract

**Background:**

Severe posttraumatic stress disorder (PTSD) may lead to non-suicidal self-injury (NSSI), and borderline personality disorder (BPD) tendencies may play a role in this process. Secondary vocational students experience more social, familial and other pressures and are more vulnerable to psychological problems. Thus, we explored the effect of BPD tendencies and subjective well-being (SWB) on NSSI in secondary vocational students with PTSD.

**Methods:**

A total of 2,160 Chinese secondary vocational students in Wuhan participated in our cross-sectional investigation. The Diagnostic and Statistical Manual of Mental Disorders, 5th edition (DSM-5), criteria for PTSD, NSSI Questionnaire, Personality Diagnostic Questionnaire-4+, subjective well-being scale, and family adaptation, partnership, growth, affection, and resolve (APGAR) Index were used. We conducted a binary logistic regression model and linear regression analysis.

**Results:**

Sex (odds ratio [OR] = 0.354, 95% confidence interval [CI] = 0.171–0.733), BPD tendencies (OR = 1.192, 95% CI = 1.066–1.333) and SWB (OR = 0.652, 95% CI = 0.516–0.824) were independent factors that predicted NSSI in secondary vocational students with PTSD. Spearman’s correlation analysis showed that BPD tendencies were positively correlated with NSSI frequency (*r* = 0.282, *P* < 0.01). SWB was negatively correlated with NSSI frequency (*r* = −0.301, *P* < 0.01). The linear regression showed that BPD tendencies (β = 0.137, *P* < 0.05 and β = −0.230, *P* < 0.001) were significantly correlated with NSSI frequency. Spearman’s correlation analysis showed that family functioning was positively correlated with SWB (*r* = 0.486, *P* < 0.01) and negatively correlated with BPD tendencies (*r* = −0.296, *P* < 0.01).

**Conclusion:**

In adolescents, PTSD in response to stressful events could lead to NSSI, and BPD tendencies promote the intensity of NSSI, while SWB diminishes its intensity. Improvement in family functioning may actively guide the development of mental health and improve SWB; such steps may constitute interventions to prevent or treat NSSI.

## 1. Introduction

Non-suicidal self-injury (NSSI), defined as intentional and self-inflicted damage to body tissues without suicidal intent, is a perplexing but common phenomenon. NSSI involves cutting, burning, biting, or scratching the skin (1). The main purpose of NSSI is to reduce negative emotions (2). The lifetime prevalence of NSSI in children and adolescents is 29.26% (3). Furthermore, in early adolescence, the prevalence of NSSI is higher in girls than in boys (4). NSSI is a significant factor threatening adolescent mental health (5). In the long run, NSSI may increase the risk of suicide (6). Thus, it is important to identify factors influencing the occurrence of NSSI to establish effective interventions for individuals with NSSI.

Compared with adulthood, adolescence is a sensitive and psychologically vulnerable period. Adolescents are more likely to be affected by traumatic events and persistent injuries experienced in the school, societal, and familial environments and are prone to high-risk behaviors (7). Some studies have shown that patients with posttraumatic stress disorder (PTSD) are at risk of developing NSSI (8). PTSD occurs after a sudden, threatening, or catastrophic life event that leads to long-term persistence of a psychiatric symptoms (9). Typical symptoms of PTSD include intrusive thoughts, avoidance of specific contexts, negative changes in cognition and mood, and increased alertness. PTSD is prone to comorbid with many mental problems, such as sleep disorders, depressive disorders, and anxiety disorders (10). Timely interventions for patients with PTSD can reduce distress, which can help to reduce the occurrence of NSSI (11).

Adolescents need interpersonal support and exposure to positive coping styles; however, adolescents with borderline personality disorder (BPD) typically express themselves in extreme ways, such as with self-harm, when faced with interpersonal problems (12). BPD is a personality disorder characterized by difficulties in interpersonal relationships, emotional instability, and impulsive behavior (13). Moreover, BPD is often present in adolescents with a history of NSSI (14). Approximately 60–75% of BPD patients have exhibited at least one self-injury behavior in their lifetime (15). Impaired emotion regulation and increased impulsivity in BPD patients are strongly associated with NSSI (2). Some studies have explored the relationship between personality subtypes and self-injury behavior in adolescents with self-injury (16). And recent studies have noted that family conflict, lack of involvement, lack of regard and other familial environment aspects promote the development of BPD in adolescents (17). Additionally, parental neglect or invalidation of children’s emotional expression may create an unsupportive familial environment that promotes the development of BPD symptoms (18).

In addition, recent studies have shown that high levels of subjective well-being (SWB), a protective factor against NSSI, can change adolescents’ strategies of coping with negative emotions, which may reduce the occurrence of NSSI (19). In particular, SWB is negatively correlated with negative emotions, and adolescents with higher SWB have fewer psychological problems (20). SWB involves positive emotion and is an overall assessment of one’s perceived quality of life. However, in the past 10 years, there has been a downward trend in SWB among adolescents (21). Therefore, improving SWB in adolescents is important for reducing the occurrence of NSSI. Family functioning, which includes cohesion, communication, family satisfaction and low family conflict, is positively correlated with the SWB of children and adolescents. Higher cohesion and good communication lead to increased happiness (22).

Accumulating evidence suggests that PTSD, SWB, and BPD tendencies are associated with NSSI. Despite these findings, to our knowledge, no studies have explored the relationships among these four variables. The relationships of NSSI with SWB and BPD tendencies in Chinese secondary vocational students has never been explored. Chinese secondary vocational students constitute a special group with a large population. Most enter secondary vocational schools because of poor academic performance (23). Thus, they are regarded by society and even their parents as “undereducated” or “failed” students; therefore, they exhibit self-depreciation and low self-esteem and are more likely to have psychological problems (24). The purpose of this study is threefold: (1) to determine the association between PTSD and NSSI in secondary vocational students and (2) to determine the associations of SWB and BPD tendencies with NSSI. We hypothesized that SWB is a protective factor against NSSI and that BPD tendencies are risk factors for NSSI. Finally, we also aimed (3) to explore the relationships of family functioning with BPD tendencies and SWB to determine potential interventions for NSSI.

## 2. Materials and methods

### 2.1. Subjects and time of survey

The research subjects were students from grade one to grade three in a secondary vocational school in Wuhan. Participants ranged in age from 15 to 18 years. To ensure the rigor and accuracy of this study, we consulted with experts in the relevant disciplines before developing and finalizing the questionnaire. A pilot survey was conducted to further refine the questionnaire. In the formal investigation, students were asked to independently complete the questionnaire onsite; the questionnaire took approximately 30 min to complete.

Investigation period: April–May 2022.

After obtaining informed consent, a total of 2,500 questionnaires were distributed to all students at the school (i.e., whole-group sampling); of these, 2,160 questionnaires were returned, for an effective rate of 86.4%. Among the participants, 992 were grade one students, 902 were grade two students and 266 were grade three students, accounting for 45.9, 41.8, and 9.5% of the sample, respectively. This study was approved by the Ethics Committee of the Affiliated Wuhan Mental Health Center, Tongji Medical College of Huazhong University of Science and Technology.

### 2.2. Research tools

#### 2.2.1. General information questionnaire

Vocational school students completed a questionnaire designed by the researchers. The questionnaire collected sociodemographic information, such as sex, grade, and family socioeconomic status.

#### 2.2.2. Primary care PTSD screen for DSM-5 (PC-PTSD-5)

The PC-PTSD was modified according to the Diagnostic and Statistical Manual of Mental Disorders, 5th edition (DSM-5), and included five topics corresponding to the five factors of re-experiencing, avoidance, hyperarousal, numbness, and negative changes in mood and cognition. Each item is scored as yes (1) or no (0); the score on all items is summed to determine the total score. A score of 3 is the threshold for the diagnosis of clinical PTSD (25). Cronbach’s α of the scale was 0.83, indicating high reliability (26).

#### 2.2.3. NSSI questionnaire

Non-suicidal self-injury was assessed in this study using one question. The question was “In the past 12 months, have you hurt yourself intentionally without attempting to commit suicide? For example, did you hurt yourself for other reasons, such as to release stress, feel better, elicit sympathy, or make something else happen?” The score of each question is 1 point for “yes” and 0 points for “no.” Participants were also asked to answer the following questions: “In the past 12 months, how many times have you hurt yourself?”, “Please indicate the main ways that you hurt yourself: (A) cutting, (B) hitting, (C) scalding/burning, or (D) other (please fill in)” and “How and where did you hurt yourself?” We provided specific questionnaires in Supplementary material.

#### 2.2.4. Personality Diagnostic Questionnaire-4+ (PDQ-4+)

In 2000, Yang Yunping and other experts to developed the Chinese version of the PDQ-4+, which is widely used in domestic research and clinical practice. This questionnaire includes 107 items to assess the pathological severity of 12 types of personality disorders (27). We selected the 11 items relevant to BPD. There are 2 sets of items, 100(6) and 101(19), and the items outside the brackets are scored. The 11th item contains 6 questions and is scored as 1 if ≥2 questions are answered with “yes” or 0 if <2 questions are answered with “yes.” Scores range from 0 to 9, with a threshold score of 5 (28). The scale has good reliability and validity in the general Chinese population, and the Cronbach’s α coefficient is 0.73–0.77 (29).

#### 2.2.5. Subjective well-being scale

The scale was developed by Andrews FM and Whitney SB in 1976 (30). Seven facial expressions (very happy, happy, relatively happy, average, relatively unhappy, miserable, and very miserable) are used to measure the respondent’s happiness and correspond to the letters A–G. Participants are asked to select the facial expression consistent with their overall perceived quality of life.

#### 2.2.6. Family adaptation, partnership, growth, affection, and resolve (APGAR) index

The family APGAR index (APGAR) was developed by Smilkstein (31). The questionnaire includes five components: adaptation, partnership, growth, affection, and resolve. There are three possible answers for each item (2 = almost always, 1 = sometimes, or 0 = hardly ever) (32). Higher scores indicate higher satisfaction with family function. Total scores of 0–3 suggest severe family dysfunction, 4–6 suggest moderate family dysfunction, and 7–10 suggest good family functioning. The Chinese version used in this study showed great reliability and validity. The Cronbach’s α values reported by studies range from 0.80 to 0.85 (33).

### 2.3. Statistical analyses

The data were analyzed using SPSS 26.0. First, we screened participants for PTSD and NSSI. Descriptive analyses of NSSI in patients with PTSD were performed, and count data are described with proportions or rates. The χ*^2^* test or precise probability method were used for group comparisons. Measurement data conforming to a normal distribution are expressed as the mean ± standard deviation (*x* ± *s*), and an independent-sample *t*-test was used to compare the two groups. Measures that did not conform to a normal distribution are expressed as the mean and interquartile range (IQR), i.e., M (*P25, P75*), and the Wilcoxon rank sum test was used for comparisons between groups. The Wilcoxon rank sum test was also used to compare the demographic characteristics of the subjects. Then, Spearman correlation analyses were performed to explore the relationships of SWB and BPD tendencies with NSSI and the frequency of NSSI. Spearman correlation analysis was also used to explore the relationships of family function and BPD tendencies with SWB. *P* < 0.05 was considered statistically significant.

## 3. Results

### 3.1. PTSD distribution among secondary vocational students

Among the 2,160 students in this study, 566 had experienced major life events or traumatic events in the past year. Among them, 241 people had PTSD, and the prevalence rate was 11% (241/2,160). The prevalence rate of PTSD in males (10.8%, 100/929) was lower than that in females (11.5%, 141/1,230). There were 122 grade one students (50.6%), 94 grade two students (39.0%), and 25 grade three students (10.4%). There were no significant differences in sex or grade between students with and without PTSD (*P* > 0.05). There was a statistically significant difference in the occurrence of NSSI among secondary vocational students (χ^2^ = 15.953, *P* < 0.05), as shown in Table 1.

**TABLE 1:** Posttraumatic stress disorder (PTSD) and sociodemographic factors of secondary vocational students in a secondary vocational school in Wuhan.

	PTSD	Without PTSD	χ^2^	*P*
Sexuality			2.829	0.093
Male	100	158		
Female	141	167		
Grade			0.056	0.973
Senior one	122	164		
Senior two	94	129		
Senior three	25	32		
Traumatic events			20.926	0
Single	183	293		
Multiple	58	32		
State of economy			7.044	0.134
Very poor	15	8		
Worse	35	36		
General	149	218		
Good	34	50		
Very good	8	13		
NSSI behaviors			15.953	0
Yes	55	34		
No	186	291		

### 3.2. NSSI characteristics in secondary vocational students with or without PTSD

There were 55 PTSD students with NSSI, and the prevalence rate of NSSI was 22.8% (55/241). Among students with PTSD, the prevalence rate of NSSI in boys (5.8%, 14/241) was lower than that in girls (17.0%, 41/241). Among PTSD students with NSSI, the most frequent family function rating was severe (65.45%). Differences in sex and family functioning between PTSD students with and without NSSI were statistically significant (*P* < 0.05). The prevalence rate of NSSI among PTSD students did not significantly vary according to grade, family socioeconomic status, or the presence of one or multiple traumatic events (all *P* > 0.05), as shown in Table 2. There were 154 non-PTSD students with NSSI, with a total prevalence rate of 8.0% (154/1919). There were significant differences in sex, age, family socioeconomic status, and the number of traumatic events between NSSI students with and without PTSD (*P* < 0.05). NSSI was detected in a total of 209 (9.68%) students. The most common method of self-injury was cutting (55.5%), and the most common self-injury site was the limbs (78.0%). Among the 55 PTSD students with NSSI, the most common mode of NSSI was cutting (63.6%), and the most common self-injury site was the limbs (81.8%).

**TABLE 2:** Non-suicidal self-injury (NSSI) and sociodemographic factors of secondary vocational students with and without PTSD in a secondary vocational school in Wuhan.

		PTSD						Without PTSD				
	NSSI		No NSSI		χ^2^	*P*	NSSI		No NSSI		χ^2^	*P*
Sexuality					6.72	0.01					27.835	<0.001
Male	14	0.058	86	0.357			35	0.018	795	0.414		
Female	41	0.17	100	0.415			119	0.062	970	0.505		
Grade					0.596	0.742					8.422	0.015
Senior one	23	0.126	108	0.59			87	0.045	783	0.408		
Senior two	3	0.016	23	0.126			52	0.027	756	0.394		
Senior three	4	0.022	22	0.12			15	0.008	226	0.118		
State of economy					3.197	0.525					11.042	0.026
Very poor	4	0.017	11	0.046			0.1	0.001	29	0.015		
Worse	10	0.041	25	0.104			23	0.012	143	0.075		
General	33	0.137	116	0.481			102	0.053	1218	0.635		
Good	8	0.033	26	0.108			26	0.014	310	0.162		
Very good	0	0	8	0.033			2	0.001	65	0.034		
Family function					11.874	0.003					0.009	9.479
Good	8	0.033	53	0.22			5	0.015	97	0.298		
Moderate	11	0.046	60	0.249			11	0.034	111	0.342		
Severe	36	0.149	73	0.303			18	0.055	83	0.255		

### 3.3. Binary regression analysis of the relationships of NSSI with BPD tendencies or SWB

Our data analysis shows that SWB is mainly concentrated in “average, relatively unhappy,” regardless of whether NSSI behavior occurs. Among NSSI students with PTSD, 23 (41.8%) of them had a average level of SWB. And 11 (20.0%) of them had a relatively unhappy level of SWB. Among non-NSSI students with PTSD, 57 (30.6%) of them had a average level of SWB. And 47 (25.3%) of them had a relatively unhappy level of SWB. Among the NSSI students, 44 (80%) of them had BPD tendencies. And among the non-NSSI students, 99 (53.2%) of them had BPD tendencies. A binary logistic regression was run with NSSI as the dependent variable, sex as a covariate, and BPD tendencies and SWB as independent variables. Variables identified as significant in the univariate regression were incorporated into the multivariate regression. BPD tendencies (odds ratio [OR] = 1.192, *P* < 0.01) were an independent risk factor for NSSI, and SWB (OR = 0.652, *P* < 0.01) was an independent protective factor against NSSI, as shown in Table 3.

**TABLE 3:** Results of binary regression analysis of the relationships of NSSI with BPD tendencies or SWB.

						95%CI	
	*B*	Standard error	Wald	*P*	OR	Lower limit	Upper limit
Sex (male)	-1.038	0.372	7.809	<0.01	0.354	0.171	0.733
BPD tendencies	0.176	0.057	9.444	<0.01	1.192	1.066	1.333
SWB	-0.427	0.119	12.844	<0.001	0.652	0.516	0.824
Constant	0.105	0.651	0.026	>0.05	1.110		

### 3.4. Spearman correlation analysis and linear regression of NSSI frequency in secondary vocational students with PTSD

Non-suicidal self-injury frequency did not exhibit a normal distribution and is described by the median (IQR). Among the 55 (22.8%) secondary vocational students with PTSD and NSSI, the median NSSI frequency in the past 12 months was 2 (1, 4). Spearman correlation analysis was used to analyze the relationships between PTSD (*M* = 4.062, SD = 0.817) and BPD tendencies (*M* = 4.160, SD = 3.38) and between SWB (*M* = 4.523, SD = 1.565) and the frequency of NSSI (*M* = 0.544, SD = 1.143). Sex was correlated with the frequency of NSSI (*r* = 0.180, *P* < 0.01). BPD tendencies were positively correlated with the frequency of NSSI (*r* = 0.282, *P* < 0.01), and SWB was negatively correlated with the frequency of NSSI (*r* = −0.301, *P* < 0.01). See Table 4.

**TABLE 4:** Results of Spearman correlation analyses of the relationships of NSSI frequency with SWB or BPD tendencies among students with PTSD (r) (*n* = 241).

	NSSI frequency	Sex	BPD tendencies	SWB
NSSI frequency	1	0.180**	0.282**	-0.301**
Sex	0.180**	1	-0.002	-0.046
BPD tendencies	0.282**	-0.002	1	-0.296**
SWB	-0.301**	-0.046	-0.296**	1

**At the 0.01 level (two-tailed), the correlation is significant.

A stepwise regression was used to select the best model according to the fit indexes and number of variables; NSSI frequency was included as the dependent variable, BPD tendencies and SWB were included as independent variables, and sex was included as the control variable. The final model exhibited good fit (*F* = 10.740, *P* < 0.01, adjusted *R*^2^ = 0.109, dependent variable standard error of prediction = 1.07961). BPD tendencies (β = 0.137, *P* < 0.05) and SWB (β = −0.230, *P* < 0.001) were independent influencing factors of NSSI frequency, as shown in Table 5.

**TABLE 5:** Results of linear regression analysis of the relationships of NSSI with BPD tendencies or SWB.

	*B*	Standard error	*Beta*	*P*	95%CI	
					Lower limit	Upper limit
Sex	0.377	0.141	0.163	<.05	0.099	-0.656
BPD tendencies	0.046	0.022	0.137	<.05	0.004	0.089
SWB	-0.168	0.047	-0.230	<.001	-0.26	-0.076
Constant	0.511	0.349		0.145	-0.177	1.199

### 3.5. Spearman correlation analyses of the relationships of family functioning with SWB and BPD tendencies in secondary vocational students with PTSD

Among secondary vocational students with PTSD, 109 (45.2%) reported severe family dysfunction, 71 (29.5%) reported moderate family dysfunction, and 61 (25.3%) reported good family functioning. Among secondary vocational students without PTSD, 101 (31.1%), 122 (37.5%), and 102 (31.4%) students reported severe family dysfunction, moderate family dysfunction, and good family functioning, respectively. Spearman correlation analyses were used to analyze the associations of family functioning (*M* = 4.403, SD = 9.337) with BPD tendencies (*M* = 4.160, SD = 3.38) and SWB (*M* = 4.523, SD = 1.565) in secondary vocational students with PTSD. Family functioning was positively correlated with SWB (*r* = 0.486, *P* < 0.01) and negatively correlated with BPD tendencies (*r* = −0.314, *P* < 0.01). Sex was not significantly correlated with SWB or BPD tendencies (*r* = −0.046 or *r* = −0.119, respectively; *P* > 0.05) (Table 6).

**TABLE 6:** Spearman correlation analysis among SWB, family functioning and BPD tendencies of students with PTSD (r) (*n* = 241).

	SWB	Family functioning	BPD tendencies	Sex
SWB	1	0.486**	-0.296**	-0.046
Family function	0.486**	1	-0.314**	-0.119
BPD tendencies	-0.296**	-0.314**	1	-0.002
Sex	-0.046	-0.119	-0.002	1

**At the 0.01 level (two-tailed), the correlation is significant.

## 4. Discussion

According to Chinese educational policies, vocational education has long been stigmatized in the field of secondary education. Most people believe that vocational education is poor quality and that secondary vocational students exhibit poor educational performance and thus must enter vocational schools. Secondary vocational students experience unfair treatment and reduced social and educational resources and may be denigrated by their families and society; thus, these individuals are at higher risk of psychological problems such as depression and anxiety (24). Secondary vocational school students have low self-esteem and negative self-perceptions (34). Unfortunately, parents are often the origin of these stereotypes, and family support and family education level are very important influences. Greater attention to the psychological state of higher vocational students is needed in China, but there are few studies on this population.

A meta-analysis of the incidence of NSSI in adolescents worldwide from 1989 to 2018 indicated a lifetime prevalence of 22.1% and a 12-month prevalence of 19.8% (35). An analysis by Sarah et al. (36) reported that the incidence rates of NSSI were 17.2% in adolescents, 13.4% in young adults and 5.5% in adults. In this survey, the prevalence rate of NSSI among students in a secondary vocational school in Wuhan in the past year was 14.0%, which differs from the above results. This difference may be due to the different NSSI measurement methods used. Use of a single yes or no question may not provide results as accurate as the use of a checklist, as individuals may not immediately recall the event for yes or no answers (37). The prevalence rate of NSSI among students with PTSD in our study was 22.9%, which was higher than that among students without PTSD. This finding is similar to that of previous studies in which PTSD was a risk factor for NSSI (38). People with PTSD have persistent negative perceptions of themselves, others, and the world and a persistent negative emotional state, which promotes the occurrence of NSSI.

In this study, the prevalence of NSSI was lower in males than in females regardless of PTSD status. This result is consistent with the findings of Bresin et al. (39) that females were significantly more likely than males to develop NSSI, possibly because of differences in sex hormones. Estrogen levels around the time of menarche increase rapidly and fluctuate dramatically in the body, and physiological systems may not be able to adapt to these changes (40). It has also been suggested that the sex difference in NSSI stem from greater susceptibility of female adolescents to negative emotions. Emotion regulation strategies differ between males and females. Females tend to use avoidance strategies (41). Previous studies have shown that adolescent NSSI peaks at the age of 15.9–17.4 years and that the incidence of NSSI decreases with age (42). This may be due to environmental changes (from middle school to secondary vocational school), maladjustment, or academic pressure. Among the students in the present study, the prevalence rate of NSSI among PTSD students was not related to grade, whereas the prevalence rate of NSSI among students without PTSD was related to grade and was highest among senior students. The effect of PTSD on NSSI may mask the effect of grade on NSSI. However, because our survey was conducted at only one school, the results may have limited generalizability. Studies have shown a higher incidence of NSSI among adolescents with low family socioeconomic status, but some studies have reported a non-significant association of these variables (43). In the present study, the incidence of NSSI among PTSD students was not related to family socioeconomic status. However, the incidence of NSSI among students without PTSD was related to family socioeconomic status, with the highest prevalence rate of NSSI among economically average students. The difference in results from those of previous studies may be due to the socioeconomic conditions in different regions, with the largest proportion of people in China having average socioeconomic status, which led to bias in the results. We demonstrated that family functioning differs between adolescents with and without NSSI. A study showed that poor family communication and lack of support may promote the development of NSSI, which in turn affects the relationship between children and parents and damages family function, forming a vicious circle (44, 45). The specific role of the familial environment in the occurrence of NSSI needs further study.

Patients with BPD are characterized by emotional instability, unstable interpersonal relationships, impulsiveness, and irritability. Patients with BPD have cognitive impairments and tend to focus on negative information (46). Adolescents with BPD have difficulties identifying and expressing emotions when dealing with interpersonal problems and often resort to extreme forms of self-harm to regulate negative emotions (12). Indeed, NSSI is one of the core symptoms of BPD, and NSSI and BPD may represent a developmental continuum (47). One study reported that compared with non-BPD individuals with NSSI, BPD individuals with NSSI are more prone to severe depression and suicidal behavior (48). Some studies have noted that people with BPD have a higher risk of NSSI than the general population (49, 50), and there is an interaction between BPD and NSSI (51). Our analysis showed that BPD tendencies are significantly positively correlated with NSSI in PTSD students, which further confirms that BPD tendencies can increase the risk of NSSI. Possible mechanisms by which BPD tendencies influence NSSI risk should be explored in future studies. Some studies have demonstrated that the probability of PTSD among patients with BPD is 30–50% and that BPD patients with PTSD are more likely to develop NSSI (52). Another study demonstrated that BPD patients use extreme methods, such as NSSI and substance use, to relieve PTSD symptoms (53). BPD tendencies and PTSD are associated with more pronounced mood dysregulation and higher risk of NSSI (54). We found that BPD tendencies were significantly associated with NSSI occurrence and positively associated with NSSI frequency in students with PTSD. BPD tendencies may lead to an increase in NSSI frequency in individuals with PTSD. Thus, symptoms of PTSD and BPD need to be addressed to reduce the risk of NSSI.

The results of this study are consistent with those of previous studies showing that SWB is a protective factor against NSSI; thus, poor SWB increases the risk of NSSI. Improving SWB may reduce the occurrence of NSSI in adolescents. Additionally, we found that family functioning significantly influenced SWB among secondary vocational students, and better family functioning led to higher SWB. Secondary vocational student with PTSD reported the largest proportion of severe family dysfunction and the smallest proportion of good family functioning. The familial environment is important for the development of emotional relationships, and a stable parent-child relationship leads to solid emotional support. Family relationships have an important influence on adolescents’ SWB, and good communication and warm relationships with family members lead to a higher level of life satisfaction (55). SWB may reflect adolescents’ social adjustment and close relationships between parents and children (56). Effective communication among family members may improve family functioning and, consequently, adolescents’ life satisfaction.

Adolescence is the period with the highest risk of PTSD and NSSI. Prevention measures focusing on adolescents after middle school graduation are important, especially those involving proper parental guidance. Based on the findings of this study, we need to pay attention to PTSD symptoms, BPD tendencies and SWB to reduce the occurrence of NSSI among adolescents. Parents’ cognitive model and emotion regulation impact children’s PTSD; additionally, parental guidance can reduce the severity of PTSD in adolescents (57). Middle school is a critical period for personality development, and good parenting at this time strengthens the parent-child bond and makes it easier for children to cultivate positive and stable mental health (58). In the field of parenting styles, parental behavior may reduce the vulnerability of children to psychological problems. Our research confirms that better family functioning reduces the risk of BPD tendencies. Based on the findings of this study, addressing PTSD symptoms, BPD tendencies, and SWB is necessary to reduce the occurrence of NSSI.

There are also some limitations in this study. First, this study is cross-sectional in design and cannot determine causal relationships among variables. Future longitudinal studies may explore whether there are differences in personality traits and SWB among adolescents with PTSD. Second, all data were self-report data (i.e., obtained by questionnaires rather than clinical interviews) and are susceptible to answer bias. Third, the focus on secondary vocational students limit the generalization of the results to other PTSD patients. Finally, when determining the factors related to NSSI among students with PTSD, we did not adjust for variables other than sex.

## Data availability statement

The raw data supporting the conclusions of this article will be made available by the authors, without undue reservation.

## Author contributions

WD, SY, and YX designed the study and developed the idea in consultation with LL, YZ, and MC. WD, SY, and ZL responsible for the review of the literature. YX, LL, and YZ extracted the data. WD performed the statistical analyses. WD, SY, and YX drafted the manuscript. LL, YZ, and MC revised the manuscript. All authors read and agreed to the published version of the manuscript.
