# Supplementary material for: Effects of BPD tendencies and subjective well-being on NSSI in adolescents with PTSD
Source: Front Psychiatry. 2023 Jun 15;14:1152352. doi: 10.3389/fpsyt.2023.1152352 (PMC10308082; doi:10.3389/fpsyt.2023.1152352)
Supplement: Supplementary file 1 [file Data_Sheet_1.PDF]

NSSI Questionnaire

1. In the past 12 months, have you hurt yourself intentionally without attempting to commit suicide? For example, did you hurt yourself for other reasons, such as to release stress, feel better, elicit sympathy, or make something else happen?.

No £0 Yes £1

1. How many times have you hurt yourself in the past 12 months? Please indicate the main ways that you hurt yourself: A) cutting, B) hitting, C) scalding/burning, or D) other (please fill in). Please indicate the main part that you hurt yourself : (A limbs 1, B trunk 2, C head or face 3 D other (please fill in).
